# “Do not detach the placenta from my baby's cord” - Lotus birth case series from Tanzania tertiary hospital

**DOI:** 10.1016/j.ijscr.2022.107630

**Published:** 2022-09-09

**Authors:** Willbroad Kyejo, Davis Rubagumya, Christian Mwalo, Lynn Moshi, Munawar Kaguta, Miriam Mgonja, Shweta Jaiswal

**Affiliations:** a Department of Family Medicine, Aga Khan University, PO Box 38129, Dar Es Salaam, Tanzania; b Department of Family Medicine, Premier Care Clinic-Masaki, PO Box 220, Dar es Salaam, Tanzania; c Department of Obstetrics and Gynecology, Aga Khan Hospital, PO Box 2289, Dar es Salaam, Tanzania

**Keywords:** Lotus birth, Umbilical nonseverance

## Abstract

**Introduction:**

Lotus birth is seldom practiced, with its prevalence being not well documented. There is no clear existing guideline or pathway for this practice. Safety in delivery as well as caring for newborn and her mother is of paramount importance. Hence, clarity for Lotus delivery in any set up is indispensable.

**Cases findings:**

We have described on case series approach to women who opted to delivery without detachment of placenta. We have described delivery characteristics, neonatal clinical course, cord, and placenta management. In year 2022 at our center, we have received two cases of lotus delivery.

**Conclusion and recommendation:**

Lotus birth is a new way of delivery in our country despite of low prevalence, it is indispensable for all cadres that deal with delivering mothers to be aware for better outcomes. It has been noted that, the risk for neonatal infection increases with this practice.

## Highlights

• Lotus birth in Tertiary hospital
• Rare case series of umbilical nonseverance
• Multidisciplinary approach

## 1 Introduction

Lotus birth is the practice of leaving the umbilical cord and placenta attached to newborn until natural detachment from the umbilicus [1]. This practice was named after Clair Lotus who observed that chimpanzee did not separate the placenta from the newborn [2]. The data on epidemiology of Lotus birth is lacking despite being practiced in several places around the world, notably with home deliveries [4].

Despite absence of scientific evidence, several reasons have been established favoring Lotus birth. Such reasons include less stress to a newborn baby while facilitating induction of immunity as well as bonding with the mother [1], [4], spiritual motivation has also been reported to favor this process [2]. There are several risks to newborn relating to the practice especially infection, while in some other areas it poses difficulties when histological examinations of the placenta are needed [1], [3].

During this kind of birth third stage of labor is managed passively with no use of either oxytocic drugs or cord traction [4]. After delivery, the placenta is washed, salted, and encased in absorbent material. Sometimes, wrapping in herbs such as lavender is done as this may aid with keeping bad odor away [4].

In African set up, Lotus birth is seldom done, since cultural beliefs involve cord severance post-delivery [5], [6]. Respecting patients opinions is one of the World Health Organization (WHO) recommendations, and this applies during maternity care [7]. In our country Tanzania there is no previous documentation on lotus birth.

In this case report we hereby report a case series of two cases in our center Aga khan hospital, Tanzania that we have seen two cases of lotus delivery. This paper has been reported in line with the SCARE 2020 criteria [8]. This article has been registered with the Research Registry with identification number researchregistry8221 and can be found through the following hyperlink Browse the Registry - Research Registry.

## 2 Case presentation

### 2.1 Case series 1

A 30-year-old female, Gravida 2 Para 0 + 1 at 38 weeks +2 days of Gestation Age, known Diabetic Type 1 on insulin since childhood, with uneventful antenatal care visits, had desired home delivery despite being counseled. Her obstetric history was remarkable for miscarriage at 8 weeks of gestation. Her social history was remarkable for being a Christian, with college education, an entrepreneur and single. Her desire for the choice of delivery was faith based and consent was solely from her. She was then brought from home in labour and on evaluation she was found to have features of Obstructed labour secondary to malpresentation. However, the cardiotocography readings were reassuring. The patient was then advised to undergo caesarean section. She consented for the procedure and agreed to neither separate her baby's cord from the placenta nor provision of Tetracycline eye ointment and Vitamin K injection to her newborn baby.

Caesarean delivery was performed by Consultant Obstetric and Gynecologist, where a 3.7 kg female baby was delivered, with Apgar score of 8 and 9 in 1st and 5th minute. With the help of nurse midwife the placenta was placed in a kidney dish, and later in a bowl that was brought by her relatives which contained salt and herbs, without being severed from the newborn. To prevent complications especially postpartum hemorrhage, oxytocin 10 IU was given after baby's delivery, and before being taken to recovery room, misoprostol 600 μg per rectal was provided.

Both mother and the baby were discharged 2 days later, with no complications, and mother refused all necessary vaccinations to be given to her baby. The mother was well informed on the signs of infection to both mother and the newborn, advised to report immediately to the hospital. On follow up, the mother reported detachment of placenta and its cord from the baby on 7th day post-delivery. Other follow up visits (2 weeks and 6 weeks post-delivery) were uneventful for both mother and her newborn.

### 2.2 Case series 2

A 36-year-old Jamaican female, Gravida 2 Para 0 Abortion 1at 39 weeks of Gestation Age, with no known comorbid, uneventful antenatal care visits, had come with the labour pain. Her obstetric history was remarkable, had previous missed abortion at 13 weeks of gestation. Her past medical history was unremarkable, had no drug or food allergy, no past surgical history. On her social history was remarkable, with history of cigarette smoking. She desires to delivery via vaginally and request her umbilical cord not to be cut because of strong spiritual belief she stated that..

“*The placenta is like a twin to the baby and nurtures him*”

in their family all her sisters had lotus delivery done from abroad. Her initial cardiotocography readings were reassuring, she delivery vaginally 3.2 kg male baby with Apgar score of 9 and 10 in 1st and 5th minute respectively. Experienced midwife and consultant obstetric perform the delivery. Post-delivery she consented for neither separate her baby's cord from the placenta nor provision of Tetracycline eye ointment and Vitamin K injection to her newborn baby which is similar as in *case 1*. Placenta was placed in a kidney dish initially, and later in her special bowl that was brought by her sisters contain herbs she only agreed on oxytocin 10 IU injection for post-partum hemorrhage control but denied on other anti-pain medications.

The mother and the baby were discharged 1 days later, with no complications, and mother refused all necessary vaccinations to be given to her baby. She was educated on danger sign and when to return for the follow up. On follow up, the mother reported detachment of placenta and its cord from the baby on 6th day post-delivery. Other follow up visits (2 weeks and 6 weeks post-delivery) were unremarkable for both mother and her newborn.

## 3 Discussion

This is a case series report on umbilical nonseverance or lotus birth. This birth practice is generally not seen in typical obstetric care, and pediatricians may not be familiar with management of this situation, which leads to inconsistent messaging to families and care teams.

**Fig. 1:**
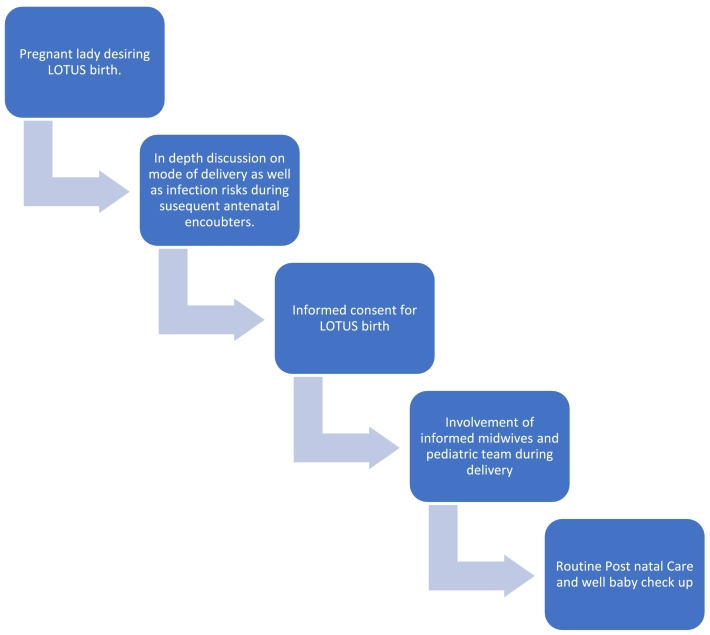
Pathway for Lotus Birth.

We had two cases over the course of 14 months in an institution with approximately 1500 deliveries/year. As it is reported in literature, LOTUS delivery is rarely encountered in an African set up [5], [6]. These two cases are the first in our center that posed stress and controversies to the department and institution at large since such a practice is not common in our country. However, it provided an experience that led to a pathway (Fig. 1) for attending such cases.

Shared decision as well as respecting patients opinion has always been encouraged provided there is no harm [7]. We have encountered special request from delivering mothers like not giving them uterotonic post-delivery, blood transfusion and vaccines, with proper protocols for such in place. However, LOTUS was new, and despite the whole process being uneventful, it is indispensable for all cadres that deal with delivering mothers to be aware for better outcomes. It has been noted that, the risk for neonatal infection increases with this practice [3], hence clear information needs to be acquired on the preparation of post-delivery care by the mother and family at home while waiting for the natural detachment.

## 4 Conclusion

Being the first case series in our setting it is important for the other institutes within our country and ministry of health which is fully responsible in policy making to address this issue in patient education programs. Our mother and newborn babies had no complication to date, but this should not be taken as the routine results. Proper counseling should be done before allowing mothers and their families mother and the family choose this mode of delivery.

## Sources of funding

This research did not receive any specific grant from funding agencies in the public, commercial, or not-for-profit sectors.

## Key clinical message

Lotus delivery pose a challenge in safety, but despite its rarity, an encounter is indispensable. With WHO advocating shared decision for delivery, a clear pathway for attending such cases is of paramount importance as far as safety for mother and her newborn is considered. This case provides a needed insight.

## Guarantor

Dr. Shweta Jaiswal, Obstetric and Gynecologist, Aga Khan Hospital

## Ethical approval

Case study is exempt from ethical approval in my institution.

## Consent

Written informed consent was obtained from the patient for publication of this case report and accompanying images. A copy of the written consent is available for review by the Editor-in-Chief of this journal on request.

## Registration of research studies

1. Name of the registry: research registry
2. Unique identifying number or registration ID: researchregistry8221
3. Hyperlink to your specific registration (must be publicly accessible and will be checked):
  Hyperlink.

## Provenance and peer review

Not commissioned, externally peer-reviewed.

## CRediT authorship contribution statement

WK: Involved in the acquisition of data, data collection, manuscript drafting and its revision.

DR: Involved in the acquisition of data, patient care-pathway design, manuscript drafting and its revision.

LM: Involved in the clinical care of the patient and manuscript revision.

MK: Involved in critical manuscript revision.

CM: Involved in clinical care of the patient and initial manuscript drafting.

MM: involved in critical manuscript revision.

SJ: Involved in the clinical care of the patient and manuscript revision.

## Declaration of competing interest

None.
